# Assessing the Relationship of Brain Metabolites to Cortical Thickness and Dementia Symptoms in Adults with Down Syndrome: A Pilot Study

**DOI:** 10.3390/brainsci14121241

**Published:** 2024-12-11

**Authors:** Katherine A. Koenig, Pallab K. Bhattacharyya

**Affiliations:** Imaging Sciences, Cleveland Clinic, Cleveland, OH 44195, USA; bhattap@ccf.org

**Keywords:** Down syndrome, magnetic resonance imaging, magnetic resonance spectroscopy, neuroanatomy, cognitive function, metabolites, cortical thickness

## Abstract

Background/Objectives: Those with the genetic disorder Down syndrome are at high risk of developing Alzheimer’s disease. Previous work shows group differences in magnetic resonance spectroscopy metabolite measures in adults with Down syndrome who have Alzheimer’s disease-related dementia compared to those who do not. In this pilot study, we assess relationships between metabolites and measures related to dementia status in a sample of adults with Down syndrome. Methods: Seventeen adults with Down syndrome were scanned using a 3 tesla MRI scanner. Magnetic resonance spectroscopy scans focused on the hippocampus and dorsal lateral prefrontal cortex. Metabolites of interest, including myo-inositol and N-acetyl-aspartate, were correlated with scores on the Dementia Questionnaire for People with Learning Disabilities, cortical thickness, and a measure of cognitive ability. In addition, cortical thickness was compared to an age- and sex-matched cohort of 17 previously scanned adults without Down syndrome. Results: Metabolite measures were not significantly related to cognitive/behavioral measures or to cortical thickness in this small cohort. Participants with Down syndrome showed widespread increases in cortical thickness compared to controls, even after accounting for potential differences in grey matter/white matter contrast. Conclusions: Metabolite values were not related to two continuous measures that have previously been associated with dementia status in those with Down syndrome.

## 1. Introduction

Early amyloid-β (Aβ) deposition is well-established in individuals with the genetic disorder Down syndrome (DS) [1] and is thought to be one of the main causes of the high rate and early onset of Alzheimer’s disease (AD) in DS (DSAD) [2]. However, variability in the severity and timing of Aβ deposition and age of dementia onset [1,3,4] suggest that other factors contribute to the development of DSAD pathology and could be used as biomarkers of AD progression or risk.

Individuals with DS show chronic inflammation throughout their lifespan [5], including early changes in peripheral and CNS inflammatory cytokine levels and microglia activation [6,7,8]. Neuroinflammation is also increasingly understood to contribute to the pathology of AD [9,10], and alterations of astroglial and microglial cells have been suggested to be involved in DSAD [11]. Myo-inositol (mI), acting as an osmolyte in glial cells, increases with both types of glial cell activation and is a possible glial activation marker [12,13,14]. DSAD is also associated with neuronal injury and loss and mitochondrial dysfunction [15,16]. Reductions in N-acetylaspartate (NAA) are related to impaired mitochondrial activity and neuronal dysfunction [17,18], suggesting that NAA is a marker of neuronal injury [19,20]. A recent meta-analysis of proton magnetic resonance spectroscopy (MRS) measures in AD and mild cognitive impairment showed consistent decreases in NAA and creatine (Cr) and increases in mI with the progression of dementia, with the largest changes occurring in the hippocampus [21,22]. Multiple studies have shown increased mI in non-demented adults with DS compared to controls without DS [23,24,25,26,27] and increased mI in DSAD compared to non-demented adults with DS [23,27,28]. Further, mI in the hippocampus was found to relate to cognitive ability in non-demented adults with DS [25]. NAA has also been reported to relate to cognitive function in adults with DS [26] and is decreased in DSAD [26,27,28]. These findings suggest that MRS measures could be developed as biomarkers of DSAD.

Here, we present the results of a pilot study designed to assess MRS measures in the hippocampus and dorsal lateral prefrontal cortex (DLPFC) in adults with DS. Both the DLPFC and the hippocampus are involved in cognitive domains that are known to show deficits in individuals with DS [29,30,31,32]. Hippocampal pathology is known to be involved in the earliest stages of AD [33] and DSAD [34], although cortical thickness in lateral prefrontal regions has been related to amyloid and tau deposition [35,36]. Given the strong association between AD stage and MRS measures in the hippocampus [21], we hypothesized that scores on a measure assessing dementia symptoms [37], the Dementia Questionnaire for People with Learning Disabilities (DLD) [38], would relate more strongly to mI levels in the hippocampus compared to the DLPFC. The goal of this study was to assess these relationships and provide sample size estimations for future work. We also assessed the relationship of mI and NAA to cortical thickness (CT), as cortical atrophy has been previously reported in DSAD [39,40] and is reported to be significantly related to NAA [27]. Finally, to add to the literature we include a comparison of CT to an age- and sex-matched sample of controls without DS.

## 2. Materials and Methods

### 2.1. Participants

Participants included 18 adults with DS, determined by clinical diagnosis. One participant (a 42-year-old female) was not able to complete any MRI scans and was excluded from further analysis. Prior to data analysis, 17 previously scanned controls without DS (CON) were selected to create an age- and sex-matched control group for the anatomical analysis. All CON scans were collected at the Cleveland Clinic on the same MRI scanner as the DS group.

Exclusion criteria for all participants included: 1. a history of major psychiatric disorder such as schizophrenia, bipolar disorder, autism, or major depressive disorder; 2. a history of neurologic diagnoses such as traumatic brain injury, stroke, or a diagnosis of seizure disorder in the past three years; 3. inability to complete study procedures; and 4. MRI-specific exclusion criteria related to claustrophobia or incompatible implants or devices. Prior to data collection, all participants were consented in accordance with the Declaration of Helsinki under protocols approved by the Cleveland Clinic Institutional Review Board. Participants with DS provided written consent or assent with written consent from a legally authorized representative as appropriate (IRB 8973, initial approval 6 August 2009). Controls provided written consent (IRB 06109, 16 February 2006; IRB 08177, 20 March 2008; IRB 13994; 25 August 2013).

### 2.2. Behavioral Testing

For participants with DS, informants completed the DLD at baseline and via phone six months later. The DLD is a 50-item questionnaire that asks informants about cognitive and behavioral changes in the person of interest, resulting in cognitive (Sum of Cognitive Scores, DLD-SCS) and social (Sum of Social Scores, DLD-SOS) scores. Higher DLD scores are typically associated with a greater decline in function, although they are recognized as relating to level of intellectual ability [37]. The DLD manual suggests longitudinal administration and recognizes an increase of ≥7 points on DLD-SCS and/or ≥5 points on DLD-SOS as indicative of a likely diagnosis of dementia [41]. Cognitive ability was assessed in those with DS using the Wechsler Adult Intelligence Scale-III (WAIS-III) [42], resulting in composite scores of verbal (VIQ), performance (PIQ), and full-scale IQ (FSIQ).

### 2.3. MRI Acquisition and Analysis

All imaging was performed on a Siemens TIM Trio 3 Tesla scanner (Siemens Healthineers, Erlangen, Germany) with a 12-channel head matrix coil. All participants underwent a whole brain T1-weighted inversion recovery turboflash (MPRAGE) with the following parameters: 120 axial slices at 1.2 mm thickness; field-of-view (FOV) 256 mm × 256 mm; matrix = 256 × 256; voxel size 1 × 1 × 1.2 mm^3^; inversion time (TI)/echo time (TE)/repetition time (TR)/flip angle (FA) = 900 ms/1.71 ms/1900 ms/8°; bandwidth (BW) = 62 kHz; time 4:03.

**Figure 1:**
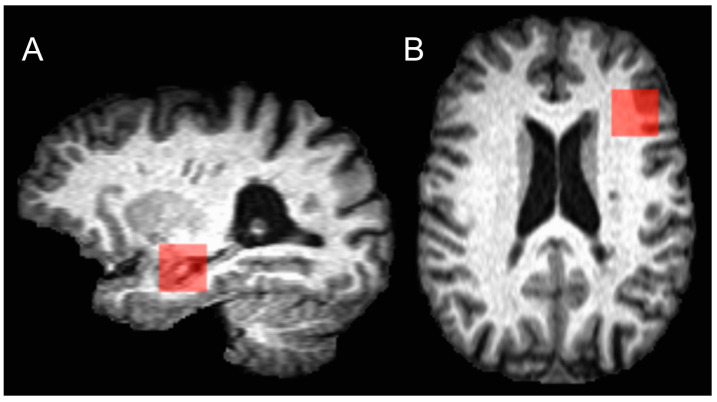
The red boxes show placement of PRESS scan voxels in a representative participant in the (**A**). hippocampus and (**B**). dorsal lateral prefrontal cortex.

Participants with DS underwent single voxel point resolved spectroscopy (PRESS) scans at the left DLPFC and hippocampus with and without water suppression (Figure 1). The parameters for the PRESS scans were TE = 30 ms; TR = 3000 ms; number of averages = 96; voxel size: 20 × 20 × 20 mm^3^ for both DLPFC and hippocampus, time 5:00. Shimming was performed using the FASTESTMAP shimming routine [43] for all spectroscopy scans. Only scans with <20 Hz full width at half maximum were accepted for further analysis.

PRESS data was analyzed using a standardized, in-house pipeline. Each voxel location was analyzed for GM, WM, and cerebrospinal fluid content using the MPRAGE segmented using the Brain Excitation Tool (BET) [44] and FMRIB’s Automated Segmentation Tool (FAST) algorithms [45] from the FSL software library [46]. PRESS data were analyzed using the MRspa software package version 1.5f, August 2018 [47], which consists of Eddy current correction (ECC2 + zero phase algorithm that performs both Eddy current and zero-order phase correction) and absolute quantification with water as internal reference using LC Model [48] fitting after applying a correction for voxel GM, WM, and CSF content for differences in relative densities of MR-visible water in different tissue types. The following metabolites were included in the basis set: L-alanine, aspartate, ascorbate, Cr, gamma amino butyric acid, glucose, gluta-mate (Glu), glutamine (Gln), glycerophosphorylcholine (GPC), glutathione (GSH), mI, scyllo-inositol, lactate, phosphocreatine (PCr), phosphorylcholine (Pcho), phosphorylethanolamine, NAA, N-acetylaspartylglutamate (NAAG), and taurine. In addition, lipid (2.0, 1.3, and 0.9 ppm) and macromolecule (0.9, 2.0, 1.2, 1.4, and 1.7 ppm) resonances were included in the basis set. Data with >20% CRLB of spectral fit were excluded from further analysis. Metabolites of interest from the LC Model output included total NAA (tNAA: NAA + NAAG), total Cr (tCr: Cr + PCr), total choline (tCho: GPC + PCho), mI, GSH and Glx (Glu + Gln).

For all participants, the MPRAGE was segmented using the Desikan–Killiany atlas [49] in the Freesurfer 7.4.1. image analysis suite [50]. Freesurfer segmentations were visually inspected for accuracy and, if necessary, corrected using methods described in the Freesurfer documentation. For each participant, mean cortical thickness (CT) was extracted for 34 regions. Mean tissue contrast between grey and white matter (GWC) was measured using a GM projection fraction of 0.3 in the same regions. Volumetric measures of interest included total and subcortical grey matter (GM), total cerebral white matter (WM), lateral ventricles, bilateral hippocampus, and WM hypointensities (WMH). With the exception of WMH, all volumes were corrected for intracranial volume (ICV) as follows: (volume/ICV) × 100.

### 2.4. Statistical Analysis

Linear correlations were used to assess the relationship of age to MRI measures and scores on the WAIS-III and DLD. Unpaired *t*-tests were used to test for sex differences. Relationships of MRS to behavioral measures were assessed using partial correlations with age as a covariate. Relationships of MRS to CT were assessed both with and without age as a covariate. The FDR was applied to the WAIS-III, DLD, and CT measures separately [51]. Power calculations were performed in G*Power 3.1.9.2 [52] using the correlations of hippocampal NAA and mI to DLD scores. Linear regression including age as a covariate was used to assess group differences in regional CT measures and ICV-corrected volumes. The false discovery rate (FDR) was applied to adjust for multiple comparisons. An exploratory analysis assessed group differences in CT using linear regression with GWC as a covariate.

## 3. Results

### 3.1. Participants

The final DS group consisted of 17 adults with DS (mean age (years) 44.9 ± 7.2, range 29–58; 7 males). The CON group consisted of 17 adults with MPRAGE data (mean age (years) 44.9 ± 7.1, range 29–58; 7 males) and was used for anatomical comparison only. As anticipated, the groups did not differ in age or sex.

### 3.2. Behavioral Measures

All participants in the DS group completed the WAIS-III and baseline DLD. FSIQ ranged from 42 to 66 (Table 1). Participants showed a wide range of scores on the DLD. DLD scores that indicate likely dementia vary depending on the baseline level of intellectual ability. Diagnosis based on a single administration of the DLD is no longer recommended [41] and we cannot be sure of the baseline level of intellectual function in the current study. To broadly characterize the dataset, we note that under the modified single administration criteria [53], two participants met the criteria for likely dementia at baseline if they were considered to have mild intellectual disability (DLD-SCS ≥ 7 and DLD-SOS ≥ 10). If these two participants were considered to have a moderate intellectual disability, they would not meet the criteria for likely dementia (DLD-SCS ≥ 25 and DLD-SOS ≥ 15). Eleven participants in the DS group had DLD scores at follow-up. Two participants had follow-up scores that may indicate dementia; one had an increase of 8 points on DLD-SCS (this person was also one of those with a score indicating possible dementia at baseline) and one had an increase of 13 on DLD-SOS. Of the remaining nine participants with follow-up scores, six did not show increases on either DLD measure and three showed an increase of ≤3 on one DLD measure. No cognitive measures showed sex differences or were significantly related to age, although the correlations of age to PIQ (r = −0.462, *p* < 0.062) and DLD-SCS (r = 0.446, *p* < 0.072) approached significance. DLD-SCS was significantly related to FSIQ (r = −0.537, *p* < 0.026) and PIQ (r = −0.604, *p* < 0.010), but DLD-SOS was not related to IQ measures.

### 3.3. MRS Measures

One participant stopped the MRI session prior to MRS scans. Of the sixteen remaining participants, all had DLPFC scans, but one was discarded from the DLPFC analysis based on the >20% CRLB rejection criterion. Three participants stopped the MRI during or just prior to the hippocampal scan. Two participants did not meet the criteria for shimming for the hippocampal scan, resulting in eleven participants with hippocampal MRS data. Of those, three were discarded based on the >20% CRLB rejection criterion. Table 2 shows the average MRS measures for both regions. Of note, metabolite values for the three participants with DLD measures that potentially indicated dementia (at baseline or follow-up) fell near the middle of the distributions of DLPFC measures. None of the three had hippocampal measures.

No MRS measures were significantly related to age or sex at the *p* < 0.05 level. Partial correlations with age were used to relate MRS measures to scores on the WAIS-III and DLD. No relationships in either the DLPFC or hippocampus survived FDR correction. Hippocampal mI and tNAA were non-significantly related to both DLD-SCS (r = −0.302, *p* < 0.510; r = −0.229, *p* < 0.622, respectively) and DLD-SOS (r = −0.184, *p* < 0.693; r = 0.162, *p* < 0.728, respectively), indicating that a minimum sample size of 229 participants for mI and 295 participants for tNAA would be required to achieve 80% power to detect a relationship to the DLD at the *p* < 0.05 level. CT measures were correlated both with and without the inclusion of age. Results were similar for both analyses, and again no relationships survived FDR correction.

### 3.4. Anatomical Measures

Due to severe atrophy, one participant in the DS group was an outlier on multiple anatomical measures (>3 standard deviations), with a thinner cortex, smaller brain volume, and larger ventricles. This participant (a 58-year-old female) and the corresponding control (a 57-year-old female) were removed from the anatomical analysis, leaving 16 participants in each group.

Both groups showed relationships of age to CT and volume measures at the *p* < 0.05 level; however, none survived FDR correction. Regardless, we elected to include age as a covariate in our analyses due to the possible impact of age-related atrophy on group differences. ICV was larger in males in both the DS (*p* < 0.032) and CON groups (*p* < 9.8 × 10^−4^). No other sex differences were found at the *p* < 0.05 level in either group.

**Figure 2:**
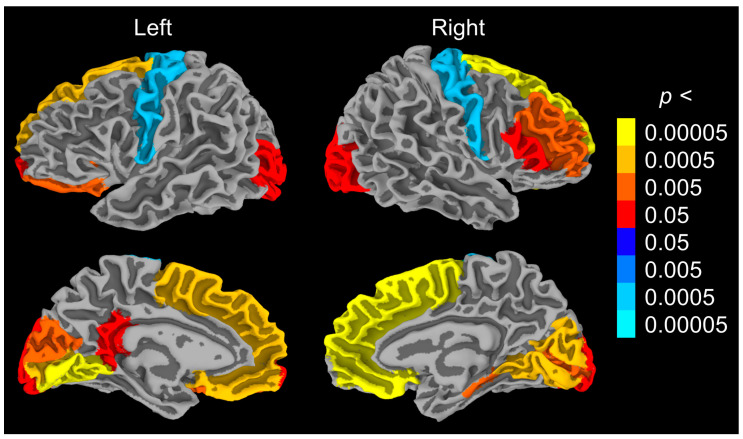
Group differences in cortical thickness in regions surviving FDR correction. Warm colors indicate DS > CON. Cool colors indicate CON > DS. CON = controls; DS = Down syndrome.

Group differences were assessed using linear regressions including group and age (Table 3; Figure 2). Compared to the CON group, the DS group showed smaller ICV (*p* < 0.002), total GM (*p* < 0.024), and bilateral hippocampal (*p* < 0.011) volumes. The DS group showed larger bilateral lateral ventricles (*p* < 0.020), increased volume of WMH (*p* < 0.003), and increased CT primarily in the frontal and occipital lobes (*p* < 0.040).

Regional GWC measures were not significantly different between groups, but all comparisons were significant for the effect of age after FDR correction (*p* < 0.030). Due to sample size restrictions and the strong correlation between GWC and age, we elected to perform an exploratory analysis of CT using regional GWC as a covariate instead of age (as opposed to including both GWC and age as covariates). With respect to group differences, the results were similar to the analysis using age, with the following exceptions: 1. right t pars triangularis thickness no longer reached significance for the full regression (*p* < 0.053); 2. left pars triangularis thickness remained significant in the full regression (*p* < 0.001) and also reached significance for the group (*p* < 0.023; DS > CON); and 3. left superior parietal thickness reached significance for both the full regression (*p* < 0.016) and the effect of the group (*p* < 0.033; DS > CON).

## 4. Discussion

This pilot study measured hippocampal and DLPFC metabolites with the goal of assessing the relationship of mI and NAA to measures related to dementia symptoms in people with DS and estimating sample size requirements for future work. Both mI and NAA are consistently reported to be altered in AD [21] and DSAD [23,26,28] and mI shows a relationship to amyloid biomarkers [27,54]. Assessment of the relationships between metabolites and individual differences in measures related to dementia progression can provide additional information about when MRS may be most useful as a biomarker.

Although numerous studies have shown differences in MRS measures between DSAD and non-demented adults with DS [26,27,28], only a few have reported relationships to cognitive measures. In a sample of 38 non-demented adults with DS, hippocampal mI was negatively related to cognitive ability [25]. NAA and Glx in the posterior cingulate were positively related to cognitive ability across a sample of 22 adults with DS and mixed dementia status [26]. Conversely, in a sample of 46 adults with DS and with mixed dementia status, hippocampal Glx did not show group differences and was not related to cognitive ability [55]. A recent study reported a positive relationship between frontal lobe NAA and IQ in 40 children with DS (mean age 10.85), suggesting that cognitive function and metabolite concentrations may be related throughout the lifespan [56]. While not a primary outcome of this study, we did not find relationships between cognitive ability, measured by the WAIS-III, and metabolite measures in either the hippocampus or the DLPFC.

Our primary measure of interest was the DLD, a commonly used instrument that has consistently shown sensitivity to cognitive decline in those with DS [37,57,58]. Based on reports of increasing mI and decreasing NAA along the disease continuum in adults with DS [27], we hypothesized that hippocampal mI and NAA would relate to DLD scores. We further hypothesized that these relationships would be strongest in the hippocampus, which shows large metabolic alterations in AD [21]. We did not see significant relationships to the hippocampus or DLPFC metabolites, either to baseline or follow-up DLD scores. The majority of our sample showed low baseline and change DLD scores, suggesting that these participants were likely to be cognitively stable. Three participants showed higher DLD scores, and, of note, these participants did not have hippocampal MRS measures. Although our small sample size may be responsible for the lack of significant relationships (particularly in the hippocampus), it is possible that the restricted range also played a role.

The inclusion of CT in this analysis was inspired by recent work showing a relationship between CT and both mI and NAA in the precuneus of adults with DS [27]. In that analysis, associations to metabolic measures were seen in the precuneus, inferior frontal cortex, and temporal cortex, and were driven by participants showing symptoms of dementia. We did not find associations between CT and metabolites, perhaps because our sample consisted primarily of those unlikely to be showing symptoms of dementia. We extended the analysis of CT by comparing the DS group to a sample of age- and sex-matched controls. A thicker cortex has been reported in both children [59] and adults [60] with DS compared to age-matched controls. Cortical thinning in those with DS has been associated with age [61] and with amyloid and tau deposition in a similar pattern to that seen in AD [35,36,62]. We elected to include GWC in our analysis, based on previous work that found differences in GWC in those with DS compared to controls and a subsequent impact on group differences in CT [60]. We did not find group differences in GWC but did find strong relationships between GWC and age, in line with previous reports [63]. Group differences in CT were similar when accounting for age or GWC, and were in line with previous reports of extensive increases in cortical thickness in those with DS in the frontal and occipital lobes and decreased thickness in the precentral gyrus [60,62]. We also found increased ventricle and WMH volumes and smaller hippocampi, all of which show changes along the DSAD continuum [64,65].

This study was designed to collect preliminary data and estimate sample sizes for future work. The most obvious limitation is the sample size, although given the relatively few MRS studies published in those with DS, the authors hope that even this preliminary work is of interest. The primary reduction in sample size came from participants that were enrolled but the scans in question were not completed. In addition to two participants who did not have any MRS scans, three participants ended the scan session prior to the completion of the hippocampal scan and two participants did not meet the criteria for an adequate shim for the hippocampal scan. We take this opportunity to encourage the reporting of incomplete or unsuccessful scans, although we realize these numbers can be difficult to determine when, for example, using a pre-existing dataset. The success rate of scans is critical to understand when evaluating the usefulness of potential biomarkers.

This study also had limited clinical measures. Diagnosis of prodromal DSAD/DSAD is often not straightforward [66] and differences in baseline intellectual ability highlight the importance of assessing changes in function [37,67]. Although we cannot be sure of the dementia status of our participants based on DLD scores alone, our supposition that the majority of our sample is cognitively stable is supported by the results of our CT comparison to controls, which are similar to those previously reported in adults with DS without dementia [60] and that had amyloid-negative imaging [62]. It is possible that the lack of MRS findings is partially due to the cognitive stability of our sample. This could indicate that longitudinal analysis is required to define meaningful differences in metabolite values prior to dementia onset, or that individual differences in metabolite values are not meaningful until later in the disease process.

## Figures and Tables

**Table 1:** Baseline behavioral data in the DS group, *n* = 17.

Measure	Mean	±	St. Dev.	Range
VIQ	55.12	±	4.9	50–66
PIQ	60.12	±	8.1	50–81
FSIQ	49.18	±	6.1	42–66
DLD-SCS	6.18	±	6.5	0–22
DLD-SOS	7.29	±	6.4	0–24

DLD-SCS = sum of cognitive scores; DLD-SOS = sum of social scores; FSIQ = full-scale IQ; PIQ = performance IQ; st. dev. = standard deviation; VIQ = verbal IQ.

**Table 2:** MRS levels (mM) in DLPFC (*n* = 15) and hippocampus (*n* = 8).

	DLPFC			HIP		
Measure	Mean	±	St. Dev.	Mean	±	St. Dev.
tNAA	9.64	±	1.07	8.28	±	1.30
tCr	6.04	±	0.79	5.98	±	0.62
tCho	1.79	±	0.31	2.13	±	0.28
mI	5.74	±	0.95	6.55	±	1.42
GSH	1.98	±	0.34	2.22	±	0.43
Glx	10.92	±	1.90	12.42	±	1.99

DLPFC = dorsal lateral prefrontal cortex; Glx = glutamate + glutamine; GSH = glutathione; HIP = hippocampus; mI = myo-inositol; st. dev. = standard deviation; tCho = total choline; tCr = total creatine; tNAA = total N-acetyl aspartate.

**Table 3:** Group differences in anatomical volume and cortical thickness measures, *n* = 16 in each group.

		Full Regression		Group	Age	Group
		F	*p*	*p*	*p*	Direction
	**Volume**					
	ICV	**7.75**	**0.0020**	0.0014	0.0957	CON > DS
	Total GM	**4.28**	**0.0234**	0.0293	0.0828	CON > DS
	Total subcortical GM	1.77	0.1887	0.6469	0.0781	
	Total WM	1.02	0.3733	0.1686	0.8439	
	Total WMH	**7.29**	**0.0027**	0.0061	0.0237	DS > CON
L	Lateral Ventricle	**5.97**	**0.0067**	0.0026	0.3255	DS > CON
R	Lateral Ventricle	**4.50**	**0.0198**	0.0074	0.4159	DS > CON
L	Hippocampus	**5.35**	**0.0106**	0.0047	0.2761	CON > DS
R	Hippocampus	**6.76**	**0.0039**	0.0021	0.1618	CON > DS
	**Cortical thickness**					
	**Frontal lobe**					
L	Superior frontal	**9.10**	**0.0009**	0.0002	0.4542	DS > CON
R	Superior frontal	**11.37**	**0.0002**	4.9 × 10^−5^	0.7511	DS > CON
L	Rostral middle frontal	2.99	0.0659	0.0348	0.2985	
R	Rostral middle frontal	**6.41**	**0.0049**	0.0014	0.5883	DS > CON
L	Caudal middle frontal	0.64	0.5351	0.4957	0.3815	
R	Caudal middle frontal	0.42	0.6600	0.4243	0.6636	
L	Pars opercularis	0.61	0.5479	0.4844	0.4047	
R	Pars opercularis	1.12	0.3407	0.3078	0.2953	
L	Pars orbitalis	**4.41**	**0.0212**	0.0963	0.0210	
R	Pars orbitalis	**3.94**	**0.0306**	0.1931	0.0190	
L	Pars triangularis	2.89	0.0718	0.7428	0.0239	
L	Lateral orbitofrontal	**9.62**	**0.0006**	0.0004	0.0727	DS > CON
R	Lateral orbitofrontal	0.35	0.7043	0.7396	0.4441	
L	Medial orbitofrontal	**10.87**	**0.0003**	0.0001	0.1763	DS > CON
R	Medial orbitofrontal	**15.52**	**2.6 × 10^−5^**	5.3 × 10^−6^	0.7154	DS > CON
L	Frontal pole	**5.82**	**0.0075**	0.0352	0.0138	DS > CON
R	Frontal pole	2.96	0.0678	0.0282	0.4394	
L	Precentral	**9.12**	**0.0008**	0.0002	0.6527	CON > DS
R	Precentral	**9.95**	**0.0005**	0.0002	0.2919	CON > DS
L	Paracentral lobule	0.42	0.6623	0.9045	0.3717	
R	Paracentral lobule	1.86	0.1741	0.3290	0.1073	
	**Cingulate**					
L	Rostral anterior cingulate	2.12	0.1380	0.0485	0.9996	
R	Rostral anterior cingulate	3.04	0.0632	0.1573	0.0541	
L	Caudal anterior cingulate	1.48	0.2437	0.1140	0.5926	
R	Caudal anterior cingulate	0.93	0.4062	0.3785	0.3079	
L	Posterior cingulate	0.12	0.8900	0.6696	0.8311	
R	Posterior cingulate	1.16	0.3288	0.5678	0.1686	
L	Isthmus cingulate	**3.72**	**0.0365**	0.0225	0.2038	DS > CON
R	Isthmus cingulate	1.92	0.1649	0.5377	0.0726	
	**Insula**					
L	Insula	1.13	0.3376	0.8328	0.1474	
R	Insula	1.41	0.2595	0.1875	0.3180	
	**Parietal Lobe**					
L	Postcentral	1.13	0.3364	0.4992	0.1887	
R	Postcentral	2.04	0.1486	0.4062	0.0756	
L	Supramarginal	3.07	0.0620	0.6676	0.0209	
R	Supramarginal	2.83	0.0753	0.6817	0.0264	
L	Superior parietal	2.36	0.1123	0.0736	0.2609	
R	Superior parietal	1.21	0.3114	0.4688	0.1774	
L	Inferior parietal	0.56	0.5793	0.5349	0.4003	
R	Inferior parietal	2.43	0.1054	0.9385	0.0356	
L	Precuneus	1.35	0.2743	0.4718	0.1492	
R	Precuneus	2.50	0.0999	0.8314	0.0339	
	**Temporal Lobe**					
L	Superior temporal	**3.96**	**0.0303**	0.5772	0.0102	
R	Superior temporal	**4.45**	**0.0206**	0.1137	0.0191	
L	Bank SSTS	1.24	0.3038	0.6478	0.1438	
R	Bank SSTS	1.94	0.1625	0.2046	0.1533	
L	Middle temporal	2.36	0.1124	0.8161	0.0394	
R	Middle temporal	2.12	0.1381	0.8803	0.0489	
L	Inferior temporal	2.53	0.0970	0.1171	0.1319	
R	Inferior temporal	2.09	0.1420	0.9470	0.0503	
L	Transverse temporal	2.16	0.1338	0.1783	0.1346	
R	Transverse temporal	**6.97**	**0.0034**	0.8355	0.0008	
L	Entorhinal	1.95	0.1603	0.9386	0.0579	
R	Entorhinal	1.86	0.1732	0.5121	0.0812	
L	Fusiform	1.39	0.2660	0.2269	0.2780	
R	Fusiform	1.80	0.1836	0.4961	0.0866	
L	Parahippocampal	0.53	0.5937	0.3885	0.5853	
R	Parahippocampal	**5.47**	**0.0096**	0.0029	0.5410	DS > CON
L	Temporal pole	**3.66**	**0.0381**	0.0682	0.0654	
R	Temporal pole	2.69	0.0850	0.0785	0.1684	
	**Occipital Lobe**					
L	Lingual	**14.62**	**4.1 × 10^−5^**	2.3 × 10^−5^	0.0548	DS > CON
R	Lingual	**10.07**	**0.0005**	0.0004	0.0546	DS > CON
L	Pericalcarine	**9.88**	**0.0005**	0.0001	0.5854	DS > CON
R	Pericalcarine	**6.29**	**0.0054**	0.0044	0.0851	DS > CON
L	Cuneus	**5.94**	**0.0069**	0.0021	0.4908	DS > CON
R	Cuneus	**12.32**	**0.0001**	0.0001	0.1177	DS > CON
L	Lateral occipital	**5.45**	**0.0098**	0.0077	0.1060	DS > CON
R	Lateral occipital	**6.36**	**0.0048**	0.0059	0.0504	DS > CON

Values in bold survived FDR correction for the full regression. Group direction = Direction of difference for measures significant for the group. CON = controls; DS = Down syndrome; L = left; R = right.

## Data Availability

De-identified data presented in this study are available on request from the corresponding author upon implementation of a Data Use Agreement.
